# Depressive symptoms among people living with HIV in Northern Tanzania: The role of family history, social support, and maladaptive coping

**DOI:** 10.1371/journal.pone.0352933

**Published:** 2026-11-05

**Authors:** Neema Allen Ng’unda, Florian Emanuel Ghaimo, Frank Kiwango, Samuel Chacha, Blandina Theophil Mmbaga, Dorothy Mushi, Ester Steven Mzilangwe

**Affiliations:** 1 Department of Psychiatry and Mental Health, KCMC University, Kilimanjaro, Tanzania; 2 Department of Psychiatry and Mental Health, Muhimbili University of Health and Allied Sciences, Dar es Salaam, Tanzania; 3 Department of Psychiatry and Mental Health, Kilimanjaro Christian Medical Centre, Kilimanjaro, Tanzania; 4 Department of Molecular Diagnostics, Sumbawanga Regional Referral Hospital, Sumbawanga, Rukwa, Tanzania; 5 Department of Pediatric and Child Health, KCMC University, Kilimanjaro, Tanzania; 6 Kilimanjaro Clinical Research Institute, Moshi, Kilimanjaro, Tanzania; Touro University California College of Pharmacy, UNITED STATES OF AMERICA

## Abstract

**Background:**

Depression is a common mental disorder among people living with HIV (PLWH) and is driven by both neuroimmunological and psychosocial pathways. In turn, depressive symptoms worsen HIV outcomes through reduced adherence, risk behaviors, and impaired immune function. This study aimed to determine the prevalence of depressive symptoms and identify associated factors among PLWH attending health facilities in northern Tanzania.

**Methodology:**

We conducted a cross-sectional study (September–October 2023) in four Care and Treatment Clinics (CTCs) across three health system levels in Moshi Municipality. Study sites were selected purposively to represent tertiary, secondary, and primary care. Within each site, adult (≥18 years) clients on ART for ≥6 months were sampled using systematic random sampling. Standardized instruments included a sociodemographic questionnaire, the Patient Health Questionnaire-9 (PHQ-9), the Duke-UNC Functional Social Support Questionnaire (FSSQ), the Brief COPE, and a 12-item HIV-related stigma scale. Data were entered and analyzed using STATA version 16. Logistic regression analysis was performed to identify associations between depressive symptoms and various independent variables.

**Results:**

A total of 598 clients participated, just above half (56%) from the secondary health facility level, and nearly three-quarters (70.9%) of participants were female. The prevalence of depressive symptoms was 14%. Participants reported having a family history of mental illness, good social support and predominantly use maladaptive coping strategies were more likely to report having depressive symptoms (AOR 2.15, 95%CI: 1.12–4.13; P 0.022), (AOR 8.19, 95%CI: 1.29–51.92; P 0.026), (AOR 3.98, 95%CI: 2.62–6.05; P < 0.001) respectively.

**Conclusion:**

Depressive symptoms are common among PLWH receiving HIV care and treatment in northern Tanzania, and are associated with a reported family history of mental illness, predominant use of maladaptive coping, and even good social support. Thus, more studies need to be done on the relationship between depressive symptoms and social support among PLWH. Integrating mental health intervention into HIV services and providing personalized interventions focused on addressing maladaptive coping and social support are key to reducing depression among people living with HIV.

## Introduction

As of 2023, an estimated 39.9 million people globally were living with HIV (PLWH), of whom approximately 30.7 million were receiving antiretroviral therapy (ART) [1]. Africa continues to bear the greatest burden of HIV infection, with approximately 26 million PLWH and 21.3 million receiving ART in 2023 [1]. In Tanzania, the prevalence of HIV among adults aged 15 years or older is estimated at 4.4%, while the prevalence in the Kilimanjaro Region of northern Tanzania was approximately 4.0% in 2023 [2]. The use of ART has significantly extended the life expectancy of PLWH [3], resulting in a 51% reduction in AIDS-related deaths globally since 2010 [4]. Consequently, HIV has increasingly become a chronic, manageable condition rather than an immediately life-threatening illness. Although ART has substantially improved virological control, survival, and physical health, attention to the psychosocial and mental health needs of PLWH remains essential [5]. Depression is one of the most common mental health conditions affecting this population and substantially diminishes quality of life, treatment adherence, and overall HIV-related health outcomes [6–8].

Globally, the prevalence of depression among PLWH is estimated at 31% [9], significantly higher than the 5% observed in the general adult population [10]. In Africa, the prevalence of depression among PLWH is estimated at approximately 36%, with rates as high as 39% reported in Eastern Africa [11]. In Tanzania, substantial variation in the prevalence of depression has been reported across populations and settings. For example, a prevalence of 9.6% was reported among PLWH in Dar es Salaam [12], 25% among pregnant women living with HIV in Kilimanjaro [13], 41% among adults newly diagnosed with HIV in Kilimanjaro [14], and 23% in a prospective observation among adults attending HIV care in Moshi [5]. Differences in study populations, clinical characteristics, duration since HIV diagnosis, measurement instruments, and health-care settings may partly explain these variations. Nevertheless, the consistently elevated burden of depressive symptoms highlights the importance of identifying modifiable and contextual factors that may contribute to depressive symptoms among PLWH.

HIV infection and depressive symptoms have a complex and multifaceted relationship through neuroimmunology and psychosocial mechanisms [14,15]. HIV infection may contribute to depressive symptoms through chronic immune activation, neuroinflammation, altered cytokine activity, and dysregulation of neurotransmitter systems involved in mood regulation [15,16]. Chronic inflammation and psychological stressors dysregulate the hypothalamic-pituitary-adrenal (HPA) axis, resulting in abnormal secretion of cortisol, which may contribute to the development or exacerbation of depressive symptoms and adversely affect immune function [16–18]. Although ART substantially reduces viral replication and improves immune function, some antiretroviral medications may cross the blood-brain barrier in the Central Nervous System (CNS), resulting in the release of proinflammatory cytokines and alteration of neurotransmitters, hence depressive symptoms [17,19,20]. In addition to these neuroimmunological effects, living with chronic infectious disease, lifelong dependence on medication, concerns about disclosure and transmission, uncertainty about the future, and experience of stigma and discrimination can generate substantial psychological distress [14,21,22]. Depressive symptoms may, in turn, negatively influence ART adherence [21] and dysregulation of the HPA-axis, which weakens the immune system, causing poor disease outcomes [23]. Also, engage in risk behaviors such as use of substances and unsafe sex, which increase HIV acquisition and worsen clinical outcomes [23–25]. These effects have also been shown in the Key population [26].

Several factors have been linked to increased depressive symptoms among PLWH. These include HIV-related stigma [27,28], insufficient social support [28,29], older age [30], maladaptive coping strategies [31–33], alcohol use [34,35], female sex [36,37], family history of mental illness [38], and longer duration of ART [14]. Importantly, these factors may interact rather than operate independently. For example, HIV-related stigma might reduce access to social support, while poor social support could limit an individual’s ability to use adaptive coping strategies when facing HIV-related stress [39,40]. Similarly, a family history of mental illness could increase vulnerability to depressive symptoms through genetic susceptibility, shared environmental exposures, and learned responses to stress [41]. Understanding these interconnected pathways is crucial for identifying individuals who may be especially vulnerable to depressive symptoms and for developing contextually appropriate interventions.

Family is particularly important in the psychosocial adjustment of PLWH because it is often the primary source of emotional, social, and financial support. Apart from genetic predisposition, caring for a parent, sibling, or other close family member with mental illness may create prolonged emotional and caregiving stress, particularly in families with limited social and economic resources [42,43]. PLWH who experience positive family relationships and adequate emotional support may be better able to mobilize adaptive coping strategies, maintain engagement in HIV care, adhere to ART, and preserve their psychological well-being and quality of life [39,44,45]. Family rejection and stigma may also contribute to internalized negative beliefs, shame, social withdrawal, and hopelessness, all of which may increase vulnerability to depressive symptoms [39,44]. Fear of family rejection and stigma may discourage PLWH from disclosing their HIV status even to close members [27,28]. Thus, the family may function either as a protective or as a predisposing factor to depressive symptoms, depending on the quality of family relationships and the individual’s experiences following HIV disclosure.

Coping strategies are another important mechanism through which PLWH respond to HIV-related stress. “Coping” refers to the cognitive and behavioural efforts individuals use to manage stressful situations and their emotional consequences [46]. The literature commonly distinguishes between problem-focused and emotion-focused coping [33,46], although coping strategies may also be broadly categorized as adaptive or maladaptive [46,47]. Adaptive coping strategies include planning, acceptance, positive reframing, active problem solving, and seeking emotional or instrumental support. These strategies may help individuals manage HIV-related challenges, improve treatment adherence, and reduce depressive symptoms [47]. In contrast, maladaptive strategies include denial, behavioral disengagement, self-blame, avoidance, and substance use, which interfere with effective problem-solving and contribute to persistent psychological distress [46,47]. Maladaptive coping and some forms of emotion-focused coping have been associated with increased depressive symptoms and poor disease outcomes [48,49]. Conversely, adaptive coping with HIV-related stigma and other disease-related stressors has been associated with better psychological well-being over time [50]. Coping strategies may also differ according to social and demographic circumstances, including gender. Evidence suggests that men may be more likely to use avoidance, denial, or alcohol as ways of managing HIV-related stress, whereas women may be more likely to use emotional responses and seek social support [46,51,52].

Perceived social support was defined as the level of support perceived by participants from family, friends, community, organizations, and coworkers [53]. Social support is closely related to both coping and depressive symptoms among PLWH. Social support among PLWH has been associated with positive adjustment, reduced behavioral disengagement, improved both physical and mental health, and better retention in HIV care [33,44]. Emotional and practical support from family members [40] or communities [52,54] can help PLWH manage HIV-related stress, treatment demands, stigma, and other stressors associated with living with chronic illness. Social support may also facilitate adaptive coping by providing individuals with information, encouragement, practical assistance, and opportunities to discuss concerns in a safe environment [55].

There is a bidirectional relationship between social support and depressive symptoms. Poor social support may increase loneliness, helplessness, and psychological distress, thereby contributing to depressive symptoms. At the same time, individuals experiencing depressive symptoms may withdraw socially, have difficulty maintaining relationships, and be less likely to seek or perceive help [33]. Thus, good social support may reduce negative psychological effects of HIV-related stigma, facilitate adaptive coping, and ultimately reduce depressive symptoms [7,27,33,54].

The high prevalence of depressive symptoms among PLWH is clinically important because depressive symptoms are associated with poor ART adherence, increased risk of unhealthy behaviors, suicide, morbidity, and reduced quality of life [24,26,56]. Despite these consequences, there is overlap between somatic symptoms of HIV and those of depression, leading to underreporting and underdiagnosis of depressive symptoms in this population [55,56]. The presence of a family member with a mood disorder, poor family dysfunction, poor social support, stigma, and poor coping with stress related to living with HIV may predispose, maintain, or precipitate depressive symptoms among PLWH [42,46,54,57,58]. However, few studies in Tanzania have looked at the association of family history of mental illness, perceived social support, coping strategies, and HIV-related stigma across primary healthcare levels to regional and tertiary care. We acknowledge that previous studies have been in and involved different sub-populations, such as pregnant women or newly diagnosed adults; however, there is limited information on depressive symptoms and the simultaneous factors assessed among PLWH. Hence, this study was conducted in HIV clinics from primary, secondary, and tertiary HIV care facilities in Kilimanjaro among PLWH on ART treatment for 6 months or above.

The study specifically aimed to: (1) determine the prevalence of depressive symptoms among PLWH; (2) assess the association between depressive symptoms and biological factors, particularly family history of mental illness and alcohol use; and (3) determine the association between depressive symptoms and psychosocial factors, including social support, coping mechanisms, and HIV-related stigma among people living with HIV attending health facilities in northern Tanzania. By examining these factors concurrently, the study seeks to provide a more comprehensive understanding of the determinants of depressive symptoms among PLWH and generate evidence to inform integrated HIV and mental health services in Tanzania.

## Methods

### Study design and setting

This hospital-based, quantitative cross-sectional study was conducted across four Care and Treatment Centers (CTCs) selected from a total of 19 CTCs in Moshi Municipality, representing different levels of the healthcare system. The selected facilities included Kilimanjaro Christian Medical Centre (KCMC), a tertiary-level hospital; Mawenzi Regional Referral Hospital (MRRH), a secondary-level facility; and two primary-level health centers, Majengo and Pasua. A multistage purposive sampling approach was employed to select the study sites. KCMC and MRRH were included as they are the only facilities at their respective levels in the municipality, while Majengo and Pasua Health Centers were chosen due to their high client volumes.

Inclusion criteria for the study were adults aged 18 years or older who had been on ART for at least six months and who provided written informed consent. Clients who were too ill to participate were excluded. Ethical approval for the study was obtained from the Muhimbili University of Health and Allied Sciences Research Ethics Committee (MUHAS-REC-07-2023-1819). In addition, permission to conduct the research was granted by the Local Authority Government and the Regional and District levels of each of the selected health facilities. Participants who scored high on the PHQ-9 questionnaire, particularly on item 9, which assesses self-harm risk, were informed of the implications of their responses and referred for appropriate mental health care at the Psychiatry Departments of either KCMC or Mawenzi, as outlined during the informed consent process.

### Sample size determination and sampling procedure

The sample size was determined based on a previous study conducted in the Kilimanjaro region of Tanzania, which reported a 23% prevalence of depression among PLWH [5]. Using Cochran’s formula [59], the initial sample size was calculated and then adjusted for a 10% non-response rate, resulting in a required sample size of 302 participants. Given the use of cluster sampling across different levels of health facilities, the sample size was further adjusted using a design effect (DEFF) [60] to account for intra-cluster variability. An intracluster correlation coefficient (ICC) of 0.005, derived from a study conducted in primary healthcare settings, was applied in the adjustment [61] This yielded a minimum required sample size of 414 participants. To enhance the statistical power of the study [62], the final sample size was increased to 600 participants**.** A systematic random sampling technique was employed to select study participants. The number of participants from each site was determined proportionally based on the total number of clients attending each facility.

### Data collection procedure and measurement tools

Data collection was conducted by NAN, in collaboration with the FEG and FK, as well as four research assistants who were trained on data collection tools. Pretesting of tools was conducted by interviewing clients from CTC for a better understanding before administration to the participants. The interviewer-administered questionnaires were conducted between 4^th^ September and 3^rd^ October 2023 at the four selected CTCs. The independent variables were assessed as sociodemographic characteristics (age, sex, marital status, education level, employment status, and study site), family history of mental illness, alcohol use, perceived social support, HIV-related stigma, and coping mechanisms. The dependent variable was the presence of depressive symptoms.

Sociodemographic data were collected using a semi-structured questionnaire that captured information such as age, sex, marital status, education level, employment status, and family history of mental illness. Employment status was defined as paid work; those working without being paid by anyone were considered unemployed, including housewives [63]. We assessed depressive symptoms using the Patient Health Questionnaire-9 (PHQ-9), a widely used and validated tool for screening, diagnosing, and monitoring depression [64]. The PHQ-9 consists of nine items assessing the frequency of depressive symptoms over the past two weeks, with each item scored from 0 (not at all) to 3 (nearly every day), resulting in a total score ranging from 0 to 27. Depression severity was categorized as follows: 0–4 (no symptoms), 5–9 (mild), 10–14 (moderate), 15–19 (moderately severe), and 20–27 (severe). A total score of 9 and above indicates having depressive symptoms. The PHQ-9 has been validated, translated into Swahili, and used in previous studies in Tanzania [5,36], with a Cronbach’s alpha of 0.83 [65].

**Perceived social support** was assessed using the Duke-UNC Functional Social Support Questionnaire (FSSQ), which includes 14 items evaluating emotional, instrumental, and social support [66]. Each item is rated on a 5-point Likert scale ranging from 1 (much less than I would like) to 5 (as much as I would like), with total scores ranging from 14 to 70. Higher scores indicate greater perceived support. For analysis, scores were categorized into tertiles: 14–23.3 (low support), 23.4–46.6 (average support), and 46.7–70 (high support). It has demonstrated good reliability, with a Cronbach’s alpha of 0.87 [67]. Although the FSSQ has not been validated in Tanzania, it has been used in similar populations [64]. In this study, the tool reliability was assessed and demonstrated good internal consistency at Cronbach’s alpha = 0.98.

**A coping mechanism was measured by the Brief COPE.** The Brief COPE Inventory consists of 28 items across 14 subscales and was used to assess coping strategies. Each item is rated on a 4-point scale from 1 (I usually don’t do this at all) to 4 (I usually do this a lot). Coping strategies were grouped into adaptive and maladaptive categories. Adaptive strategies included active coping, planning, positive reframing, acceptance, humor, religion, and the use of emotional and instrumental support, with a total score of 64. Maladaptive strategies included self-distraction, denial, venting, substance use, behavioral disengagement, and self-blame, with a total score of 48. Scores were categorized as high or low based on the extent of use [66]. Although the tool has not been validated in Tanzania, the tool’s reliability was assessed and demonstrated good internal consistency at Cronbach’s alpha = 0.92.

HIV-related stigma was defined as the participant’s perceived or experienced negative attitudes, discrimination, or social consequences associated with HIV infection [27]. It was measured using Berger’s HIV Stigma Scale [67], which includes 12 items covering four domains: personalized stigma, disclosure concerns, public attitudes, and negative self-image. Each item is rated on a 4-point Likert scale from 1 (strongly disagree) to 4 (strongly agree), yielding a total score ranging from 12 to 48. A score of 30 or higher indicated high perceived stigma [68]. This tool has been validated in Tanzania and has demonstrated good construct validity and reliability, with a Cronbach’s alpha exceeding 0.70 [69].

### Data processing and analysis

Data were collected using REDCap and analyzed with STATA version 16. Descriptive and inferential statistics were used to summarize and interpret the data, with results presented in tables and figures. Categorical variables were summarized using frequencies and percentages, while continuous variables were described using means and standard deviations. Bivariate and multivariate logistic regression analyses were conducted to examine associations between depressive symptoms and independent variables. Statistical significance was set at p < 0.05, with 95% confidence intervals (CIs). For bivariate analysis, crude odds ratios (COR) were reported, and variables with a p-value less than 0.20, such as age, employment status, family history of mental illness, alcohol use, social support, perceived HIV stigma, adaptive coping, and maladaptive coping mechanisms, were adjusted for potential confounders in multivariate analysis. The final multivariate results were reported as adjusted odds ratios (AORs) with 95% confidence intervals (CIs), and statistical significance was set at p < 0.05. We used weights to account for unequal selection probabilities and adjusted for clustering and stratification while calculating the prevalence of depression and fitting regression models.

## Results

### Sociodemographic and psychosocial characteristics of the study participants

A total of 600 participants were recruited from three levels of healthcare facilities, achieving a 100% response rate. Of these, 598 participants were included in the final analysis after excluding two individuals due to incomplete data. Just over half of the participants (56%) were recruited from secondary-level health facilities. The majority were female (70.9%), and more than half (58.4%) were aged between 40 and 59 years, with a mean age of 48.3 years (SD ± 12.7). Regarding marital status, 40.7% of participants reported being married or cohabiting. In terms of education, 68.1% had either informal or primary-level education. Nearly one-third (29.6%) of the participants were unemployed. A family history of mental illness was reported by 7.86% of participants, while 34.4% reported a history of alcohol use.

56.86% and 77.1% of participants reported poor social support and low perceived HIV-related stigma, respectively. Moreover, 53.5% and 87.3% of participants reported using predominantly low levels of adaptive and maladaptive coping strategies. Details of these results are reported in Table 1.

**Table 1:** Sociodemographic and Psychosocial Characteristics of the PLWH by site in northern Tanzania (N = 598).

Variable	Tertiary health facility N = 186 n (%)	Secondary health facility N = 335 n (%)	Primary health facility N = 77 n (%)	Total n (%)
Sex				
Male	58(33.33)	99(56.90)	17(9.77)	174 (29.1)
Female	128(30.19)	239(55.66)	60(14.15)	424 (70.9)
Age in groups Mean (SD)=48.3(±12.7)				
18-39	28(20.44)	84(61.31)	25(18.25)	137 (22.9)
40-59	114(32.66)	191(54.73)	44(12.61)	349 (58.4)
60>	44(39.29)	60(53.57)	8(7.14)	112 (18.7)
Marital Status				
Single	30(25.86)	72(62.07)	14(12.07)	116 (19.4)
Divorced/Widowhood	66(27.62)	149(62.34)	24(10.04)	239 (40.0)
Married/Cohabiting	90(37.04)	114(46.91)	39(16.05)	243 (40.6)
Education level				
Informal/primary	135(33.17)	218(53.56)	54(13.27)	407 (68.1)
Secondary	42(28.97)	87(60.00)	16(11.03)	145 (24.2)
College/Higher	9(19.57)	30(65.22)	7(15.22)	46 (7.7)
Employment status				
Unemployed	52(29.38)	100(56.50)	25(14.12)	177 (29.6)
Employed	134(31.83)	235(55.82)	52(12.35)	421 (70.4)
Family history MI				
No	172(31.22)	307(55.72)	72(13.07)	551 (92.1)
Yes	14(29.79)	28(59.57)	5(10.64)	47 (7.9)
Alcohol use				
No	117(30.00)	227(58.21)	46(11.79)	390 (65.6)
Yes	67(32.68)	107(52.20)	31(15.12)	205 (34.4)
Social support				
Poor (14–23.3)	82(24.12)	216(63.53)	42(12.35)	340 (56.9)
Average (23.4–46.6)	93(44.93)	83(40.10)	31(14.98)	207 (34.6)
Good (46.7–70)	11(21.57)	36(70.59)	4(7.84)	51 (8.5)
Perceived HIV stigma				
Low (score <30)	158(34.27)	240(52.06)	63(13.67)	461 (77.1)
High (score ≥30)	28(20.44)	95(69.34)	14(10.22)	137 (22.9)
Adaptive coping mechanism				
Low (score<32)	105(32.81)	173(54.06)	42(13.13)	320 (53.5)
High (score≥32)	81(29.14)	162(58.27)	35(12.59)	278 (46.5)
Maladaptive coping mechanism				
Low (score<24)	169(32.38)	290(55.56)	63(12.07)	522 (87.3)
High (score≥24)	17(22.37)	45(59.21)	14(18.42)	76 (12.7)

KEY: SD = standard deviation

### Prevalence of depressive symptoms among PLWH

Among the 598 participants included in the final analysis, 14% (n = 86) reported experiencing depressive symptoms. Within this group, 5% exhibited moderate symptoms, 4% had moderately severe symptoms, and 1% reported severe depressive symptoms. These findings are illustrated in Fig 1.

**Fig 1:**
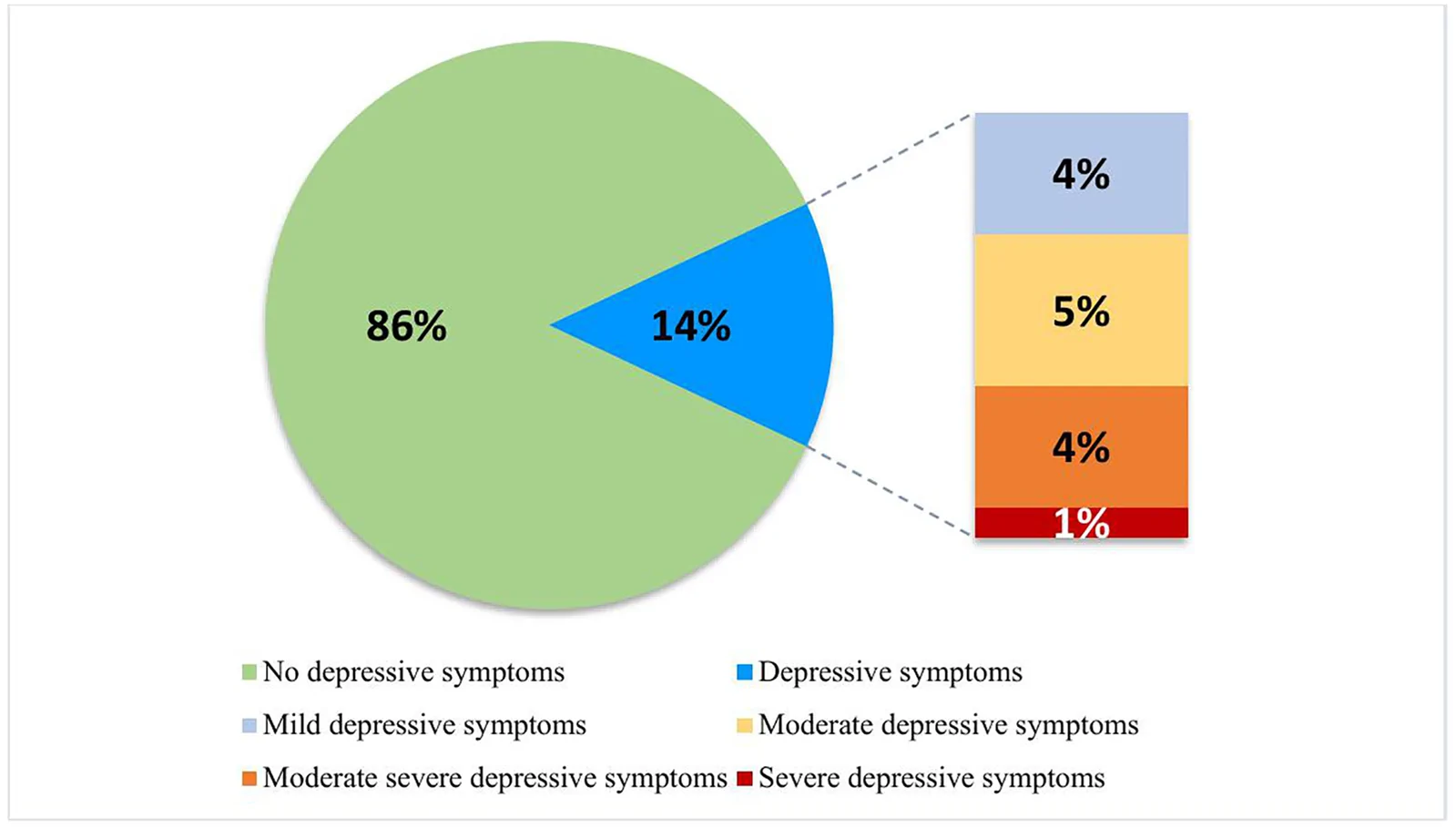
Prevalence of depressive symptoms.

### Factors associated with depressive symptoms among PLWH

Associations between each independent variable and depressive symptoms were first tested using the chi-square test, and unadjusted odds ratios were then estimated using binary logistic regression. Age group was statistically significant, with higher odds of depression for participants aged 40−59 than other age groups (COR = 1.64; 95% CI: 1.17–2.32; p = 0.004). Employed participants were 18% less likely to be depressed than unemployed participants (COR = 0.82; 95% CI: 0.70–0.95; p = 0.007). Participants who reported having a family history of mental illness were three times more likely to be depressed than those with none (COR = 3.27; 95% CI: 1.29–8.33; p = 0.013). Good social support had ten times higher odds of depression than average or poor social support (COR = 10.12; 95% CI: 2.42–42.27; p = 0.002). Participants with higher perceived stigma were more likely to be depressed (COR = 1.98; 95% CI: 1.12–2.70; p=<0.001). For coping mechanisms, participants who used adaptive coping were almost two times more likely to be depressed (COR = 1.74; 95% CI: 1.12–2.70; p = 0.013), and those with higher maladaptive coping were three times more likely to be depressed (COR = 3.55; 95% CI: 3.46–3.65; p = < 0.001). See Table 2. All of the variables with p < 0.20 were included in the multivariable logistic regression model.

**Table 2:** Bivariate analysis of factors associated with Depressive Symptoms among PLWH in Moshi Municipal.

Variable	Total	Reported Depression	COR (95% CI)	P
Site Level				
Primary	77	10 (13.0)	0.88 (0.40-1.92)	0.746
Secondary	335	47 (14.0)	0.96 (0.58-1.60)	0.879
Tertiary	186	27 (14.5)	Ref	
Sex				
Female	424	62 (14.6)	1.18 (0.88-1.60)	0.269
Male	174	22 (12.6)	Ref	
Age group, years				
18–39	137	14 (10.2)	Ref	
40–59	349	55 (15.8)	1.64(1.17-2.32)	0.004
≥60	112	15 (13.4)	1.36 (0.75-2.47)	0.315
Marital status				
Single	116	17 (14.7)	Ref	
Divorced/Widowhood	239	36 (15.1)	1.03 (0.62-1.71)	0.900
Married/Cohabiting	243	31 (12.8)	0.85 (0.35-2.03)	0.717
Education level				
Informal/primary	407	57 (14.0)	Ref	
Secondary	145	19 (13.1)	0.93 (0.67-1.28)	0.643
College/Higher	46	8 (17.4)	1.29 (0.66-2.53)	0.453
Employment status				
Unemployed	177	28 (15.8)	Ref	
Employed	421	56 (13.3)	0.82 (0.70-0.95)	0.007
Family history MI				
No	551	69 (12.5)	Ref	
Yes	47	15 (31.9)	3.27 (1.29-8.33)	0.013
Alcohol Use				
No	390	48 (12.3)	Ref	
Yes	205	36 (17.6)	1.52 (0.89-2.59)	0.126
Social support				
Poor	340	22 (6.47)	Ref	
Average	207	41 (19.81)	3.57 (0.86-14.83)	0.080
Perceived HIV stigma				
Good	51	21 (41.18)	10.12(2.42-42.27)	0.002
Low	461	55 (11.9)	Ref	
High	137	29 (21.2)	1.98 (1.78-2.20)	0.000
Adaptive coping mechanism				
Low	320	35 (10.9)	Ref	
High	278	49 (17.6)	1.74 (1.12-2.70)	0.013
Maladaptive coping mechanism				
Low	522	60 (11.5)	Ref	
High	76	24 (31.6)	3.55 (3.46-3.65)	0.000

Key: CI = Confidence Interval, COR = Crude Odds Ratio.

Table 3 represents the multivariate analysis, which revealed several factors which were significantly associated with depressive symptoms among people living with HIV. Participants with a family history of mental illness were more than twice as likely to experience depressive symptoms compared to those without such a history (AOR = 2.15; 95% CI: 1.12–4.13; p = 0.022). Interestingly, individuals who reported good social support were found to have a significantly higher likelihood, over eight times, of experiencing depressive symptoms (AOR = 8.19; 95% CI: 1.29–51.92; p = 0.026). Additionally, participants who employed high levels of maladaptive coping mechanisms were nearly four times more likely to report depressive symptoms compared to those with low levels of maladaptive coping (AOR = 3.98; 95% CI: 2.62–6.05; p < 0.001).

**Table 3:** Multivariate analysis of factors associated with Depressive Symptoms among PLWH in Moshi Municipal.

Variable	AOR (95% CI)	Adjusted P-value
Age group, years		
18–39	Ref	
40–59	1.61(0.85-3.06)	0.146
≥60	1.31(0.65-2.64)	0.451
Employment status		
Unemployed	Ref	
Employed	0.97(0.73-1.30)	0.847
Family history MI		
No	Ref	
Yes	2.15(1.12-4.13)	0.022
Alcohol Use		
No	Ref	
Yes	1.10(0.88-1.38)	0.396
Social support		
Poor	Ref	
Average	4.06(0.99-16.78)	0.052
Good	8.19(1.29-51.92)	0.026
Perceived HIV stigma		
Low	Ref	
High	1.09(0.77-1.53)	0.642
Adaptive coping mechanism		
Low	Ref	
High	1.36(0.88-2.10)	0.162
Maladaptive coping mechanism		
Low	Ref	
High	3.98(2.62-6.05)	<0.001

Key: CI = Confidence Interval, AOR = Adjusted Odds Ratio, Adj. P=Adjusted P value.

## Discussion

The present study examined the prevalence of depressive symptoms and the association between depressive symptoms and biological factors and psychosocial factors among PLWH attending different levels of health facilities. This study found that depressive symptoms are common among PLWH attending CTCs in northern Tanzania, highlighting a significant mental health burden within this population. Moreover, depressive symptoms are more likely to be reported by PLWH who have a family history of mental illness, predominantly use high levels of maladaptive coping strategies, and perceive good social support. These findings underscore the complex interplay of biopsychosocial factors influencing mental health among PLWH.

The prevalence of depressive symptoms observed in this study is higher than that of the general population. This could be explained by the stress due to the chronicity of the disease [70]. This finding is lower compared to a multi-country study in Tanzania, Kenya, and Namibia reporting a 28% prevalence [68]. The lower rate in this study may be due to differences in assessment tools (PHQ-9 vs. CES-D). Similarly, a previous study in Kilimanjaro found a 41% prevalence among newly diagnosed individuals [36]. This may suggest that being on HIV care and treatment for a substantial period of time may facilitate the reduction of HIV-associated physical and mental health conditions and enhance adjustment and acceptance of their diagnosis [7,71,72]. Also, the prevalence of this study is lower than that reported in other sub-Saharan countries, such as Ethiopia, Uganda, and South Africa. This difference can be explained by social cultures. These findings show clinical implications for routine screening for depression among PLWH and emphasize the need to integrate mental health services within HIV care to improve overall treatment outcomes and quality of life.

PWLH who reported having a family history of mental illness were more likely to experience depressive symptoms, consistent with findings from studies in a nearby Eastern African country [41,73]. This association may be explained by not only genetic predisposition but also environmental influences such as poor family functioning and poor emotional support within the household among PLWH [39,58]

Participants with good social support were more likely to report experiencing depressive symptoms compared to those with poor support. This counterintuitive finding should be interpreted with caution. First, FSSQ, although widely used, has not been formally validated in the Tanzanian context among PLWH, and the wide confidence intervals observed suggest imprecision in the estimated association. These factors limit the conclusiveness of the findings, hence the need to validate this tool in further research.

The observed association in this study may reflect that individuals experiencing depressive symptoms are more likely to seek or receive social support [33,52], or that the support received was perceived as inadequate or indifferent, potentially exacerbating their distress [74]. Additionally, the FSSQ measures general functional social support, emotional and confidant support, and it does not specifically assess HIV-related social support. As a result, the findings may reflect general social support rather than support specifically linked to living with HIV, which may be more relevant for mental health outcomes among PLWH.

This study contrasts with most studies, which report that strong social support reduces the risk of depression among PLWH [28,75,76]. The discrepancy may be due to differences in measurement tools; this study used the FSSQ, which focuses on perceived social support, whereas others used the Oslo Social Support Scale, which assesses general perceived social support. Additionally, a study in Kilimanjaro using the FSSQ found no association [36], possibly due to differences in how social support levels were categorized into quartiles in the previous study versus tertiles in the current study.

PLWH use various coping strategies to manage the psychological stress associated with chronic illness, stigma, and lifelong treatment [45].In this study, individuals who relied heavily on maladaptive coping mechanisms were more likely to experience depressive symptoms. This may be because maladaptive coping among PLWH may be amplified by HIV related stressors, which increase reliance on avoidance, denial, or disengagement, which can undermine psychological adjustment and exacerbate depressive symptoms [66]. These findings align with studies from India [34] and Cameroon [49], but differ from a study in Nigeria, where PLWH reported higher use of problem-focused coping [66]. Such differences may stem from cultural and contextual variations in coping behaviors. Overall, these findings highlight the importance of evaluating coping strategies within the specific psychosocial context of living with HIV rather than assuming they are the same as in the general population.

Although other studies in Africa have identified factors such as old age [77], female sex [29,68], marital status [29,76], unemployment [29], lower education level [78], alcohol use [68,79], and perceived HIV stigma [77,80] as significant predictors of depression, these variables were not significantly associated with depressive symptoms in this study. The difference may be explained by differences in culture, tools used, and settings where the study was conducted.

### Implications of the findings on clinical practices

The findings of this study emphasize routine screening for depressive symptoms, as it’s common among PLWH. Routine screening for depressive symptoms is important among PLWH for early identification, treatment, and referral to higher levels of care for affected individuals [81]. Similarly, studies in Sub-Saharan Africa encourage integration of mental health screening in HIV care, even with limited numbers of mental health specialists, and be effective [81,82]. Healthcare providers in HIV care can be trained on how to screen, manage, and when to refer individuals with depressive symptoms [83]. Incorporating psychosocial interventions that support families [84,85], address depressive symptoms [86], stigma [87] and build resilience [86] in HIV care has been shown to increase treatment adherence, reduce depression and improve quality of life among PLWH.

### Implications of the findings in Public Health Interventions

Integrating mental health education into HIV programs and the community to raise mental health awareness and reduce stigma on HIV and mental disorders. Mental health education has been shown to increase knowledge, change attitudes and practices towards mental disorders and HIV care [88,89]. Development of technology and increased access to digital platforms for different groups of people has led to the development of digital mental health interventions that help in the screening and treatment of depressive symptoms, even in HIV care, to address the burden of depressive symptoms and improve HIV care [90–92].

### Implications of the findings for future research

Future research should be done to establish the temporal relationship between psychosocial factors and depressive symptoms among PLWH, also exploring deeper insights into the lived experience of depressive symptoms, social support, and coping mechanisms. Intervention studies are also needed to evaluate the effectiveness of family-based support programs, psychosocial counseling, and coping skills interventions in reducing depression in Tanzanian settings, as many studies have been conducted in other Sub-Saharan countries and have been shown to be effective [84,85].

### Study limitation

These study findings should be interpreted in light of some limitations. This study is based on self-reported information that may be predisposed to social desirability and recall bias. Therefore, it could lead to participants underreporting or overreporting of depressive symptoms. To mitigate this, the research team emphasized the study’s purpose and confidentiality. Additionally, the Brief COPE and Duke-UNC Functional Social Support tools have not been validated in Tanzania, and wider confidence intervals for social support may affect the accuracy of the findings. Most of the participants were female, which may influence the observation of psychosocial patterns, especially social support. Lack of data on duration of living with HIV is a notable limitation, as time since diagnosis may influence adaptation, coping, and support networks, which may be a potential confounding factor. The majority of the participants were middle-aged, and the study included those on treatment for 6 months or more; the findings may not be generalizable in old age, adolescents, or those newly diagnosed or with loss of follow-up. Although certain limitations were present, the study met its targeted sample size and included representation from all levels of healthcare facilities, enhancing the robustness and generalizability of the conclusions.

## Conclusion

Findings of this study suggest that depressive symptoms affect a significant proportion (14%) PLWH in northern Tanzania, where factors like family history of mental illness, high use of maladaptive coping strategies, and unexpectedly good perceived social support increased the risk of depressive symptoms. These findings highlight the complex and sometimes counterintuitive nature of mental health determinants in this population. The results underscore the importance of integrating mental health screening and support into HIV care. Further longitudinal and qualitative research is needed to explore how social support from families, peers, and the community influences the development, management and outcome of depressive symptoms among PLWH. More studies on the effectiveness of family-centred interventions and other social support interventions that improve depressive symptoms among PLWH are needed, which can be integrated into HIV routine care, especially for those with a family history of mental illness. Additionally, interventional studies that aimed to reduce stigma and maladaptive coping by employing more adaptive coping should be employed to address depressive symptoms in this population.

## Supporting information

S1 Data Data for depressive symptoms among people living with HIV in Northern Tanzania: the role of family history, social support, and maladaptive coping. (DTA)
